# Molecular Characterization of NAD^+^-Dependent DNA Ligase from *Wolbachia* Endosymbiont of Lymphatic Filarial Parasite *Brugia malayi*

**DOI:** 10.1371/journal.pone.0041113

**Published:** 2012-07-16

**Authors:** Nidhi Shrivastava, Jeetendra Kumar Nag, Shailja Misra-Bhattacharya

**Affiliations:** Division of Parasitology, Central Drug Research Institute, Lucknow, Uttar Pradesh, India; Louisiana State University and A & M College, United States of America

## Abstract

The lymphatic filarial parasite, *Brugia malayi* contains *Wolbachia* endobacteria that are essential for development, viability and fertility of the parasite. Therefore, wolbachial proteins have been currently seen as the potential antifilarial drug targets. NAD^+^-dependent DNA ligase is characterized as a promising drug target in several organisms due to its crucial, indispensable role in DNA replication, recombination and DNA repair. We report here the cloning, expression and purification of NAD^+^-dependent DNA ligase of *Wolbachia* endosymbiont of *B. malayi* (*w*Bm-LigA) for its molecular characterization. *w*Bm-LigA has all the domains that are present in nearly all the eubacterial NAD^+^-dependent DNA ligases such as N-terminal adenylation domain, OB fold, helix-hairpin-helix (HhH) and BRCT domain except zinc-binding tetracysteine domain. The purified recombinant protein (683-amino acid) was found to be biochemically active and was present in its native form as revealed by the circular dichroism and fluorescence spectra. The purified recombinant enzyme was able to catalyze intramolecular strand joining on a nicked DNA as well as intermolecular joining of the cohesive ends of BstEII restricted lamda DNA in an *in vitro* assay. The enzyme was localized in the various life-stages of *B. malayi* parasites by immunoblotting and high enzyme expression was observed in *Wolbachia* within *B. malayi* microfilariae and female adult parasites along the hypodermal chords and in the gravid portion as evident by the confocal microscopy. Ours is the first report on this enzyme of *Wolbachia* and these findings would assist in validating the antifilarial drug target potential of *wBm-*LigA in future studies.

## Introduction

More than 1.3 billion people in 81 countries worldwide are threatened by lymphatic filariasis (LF). The current antifilarial treatments e.g. diethylcarbamazine, ivermectin and albendazole interrupt the cycle of transmission of the causative filarial parasites *Wuchereria bancrofti, Brugia malayi* and *B. timori*, by predominantly killing the microfilariae (mf). However, a little adulticidal effect allows adult worms to survive in human hosts for up to decades. The current treatments have to be administered annually on a community wide basis for several years to break transmission of infection and this may instigate emergence of drug resistance. There is an urgent need to develop novel drug target/s particularly pertaining to adult parasites. With the discovery of intracellular endosymbiont alphaproteobacteria, *Wolbachia*, and the experimental evidence showing its indispensable role in parasite survival, development and embryogenesis [1], [2], makes *Wolbachia* an attractive antifilarial drug target. Furthermore, *Wolbachia* have been identified as a major contributor of the inflammatory host pathology and severe adverse reactions to antifilarial chemotherapy. Therefore, characterization of novel proteins of *Wolbachia* is a prerequisite to identify the factors responsible for pathogenesis of the disease, understanding the host- parasite interactions and developing new protein/enzyme targets for antifilarial chemotherapy. A few proteins of *Wolbachia* have been identified and their functions are being investigated such as *Wolbachia* surface protein (WSP), heat shock protein 60 (HSP60), independent phosphoglycerate mutase (iPGM) and the enzymes involved in Heme biosynthetic pathway. The sequenced genome of the *Wolbachia* endosymbiont of *B. malayi* (*w*Bm) offers an unprecedented opportunity to identify new *Wolbachia* drug targets [3]. DNA ligases are indispensable for many fundamental processes in DNA metabolism including the linkage of Okazaki fragments during replication, recombination processes and repair pathways requiring resynthesis of DNA [4], [5]. Their crucial function is emphasized by the fact that the eukaryotic cells contain several isoenzymes and that the viruses encode their own ligases [6]. They are grouped into two families depending on their cofactor requirement, one is NAD^+^-dependent DNA ligase requiring NAD^+^ as a cofactor (present in bacteria and eukaryotic viruses) and the other is ATP-dependent DNA ligase requiring ATP as nucleotide cofactor (present in eukaryotes) [7], [8]. NAD^+^-dependent DNA ligases have been characterized in several organisms such as *Ferroplasma* [9], *Mycobacterium tuberculosis* [10], *Escherichia coli* [11], *Amsacta moorei entomopoxvirus* [12] and *Staphylococcus aureus* [13]. Due to its important role in nuclear metabolism, this enzyme is being exploited as a potential antibacterial drug target in *M. tuberculosis*, *E. coli, Salmonella enterica serovar Typhimurium, Bacillus subtilis* and *S. aureus* [14], [15], [16]. Therefore, an attempt was made to clone, overexpress and purify NAD^+^-dependent DNA ligase from *Wolbachia* endosymbiont of *Brugia malayi* (*w*Bm-LigA) with an aim to functionally characterize it. To the best of our knowledge, this is the first ever report on the characterization of any nuclear metabolic enzyme from an obligate mutualist alphaproteobacterium, *Wolbachia* of filarial parasite.

## Results

### ∼75 kDa recombinant *w*Bm-ligA was over-expressed and purified

**Figure 1:**
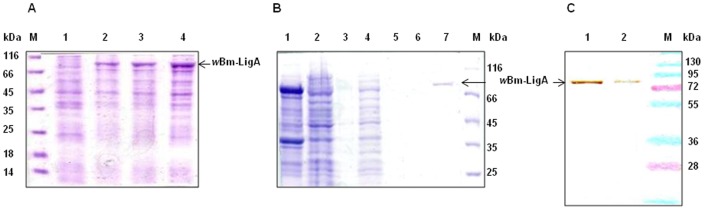
Overexpression, purification and Western blot analysis of recombinant *w*Bm-LigA. (A) Overexpression of *w*Bm-LigA in Rosetta *E. coli* cells**.** lane M, standard protein molecular weight marker; lane1, total proteins extracts isolated from the uninduced *E. coli* cells; lanes 2–4, total proteins extracts isolated from the *E. coli* cells induced with 0.2mM, 0.5mM and 1.0mM of isopropyl-1-thio-β-D-galactopyranoside (IPTG). ∼20 µg of proteins were loaded per lane. (B) Purification of *w*Bm-LigA by affinity chromatography using nickel column. lane 1, soluble *E. coli* proteins following induction with 1.0 mM IPTG at 25°C for 5 h; lane 2, flow through from the nickel column; (lanes 3–6), washes from column prior to elution; lane 7, elution of His-tagged purified recombinant *w*Bm-LigA) at 250 mM imidazole concentration. 5 µg of recombinant protein was loaded onto the gel; lane M, standard protein molecular weight marker. (C) Western blot analysis. lane1, confirmation of His-tagged *w*Bm-LigA expression with reaction of 20 µg of recombinant protein with anti-His antibody; lane 2, reaction of 10 µg of *w*Bm-LigA protein with anti-His antibody; lane M, standard protein molecular weight marker.

*w*Bm-ligA gene was amplified from the genomic DNA of adult *B.malayi* worms and the amplified product was successfully cloned in pTZ57R/T vector for maintenance. Further, *w*Bm-ligA gene was subcloned in pET28a(+) and confirmed by restriction digestion of recombinant construct of pET28a-ligA. *w*Bm-LigA was expressed as a ∼75 kDa protein with (His)6-tag fused at the N and C-terminus in Rosetta *E. coli* cells (Figure 1A). The recombinant protein was obtained in the soluble form and purified by affinity chromatography on a Ni-Nitrilotriacetic agarose column (Ni-NTA). The recombinant protein was eluted at 250 mM imidazole (Figure 1B) and finally confirmed with anti-His antibody in the Western blot (Figure 1C).

### Conserved domain architecture of *w*Bm-LigA showing phylogenetic divergence from eukaryotic DNA ligases

**Figure 2:**
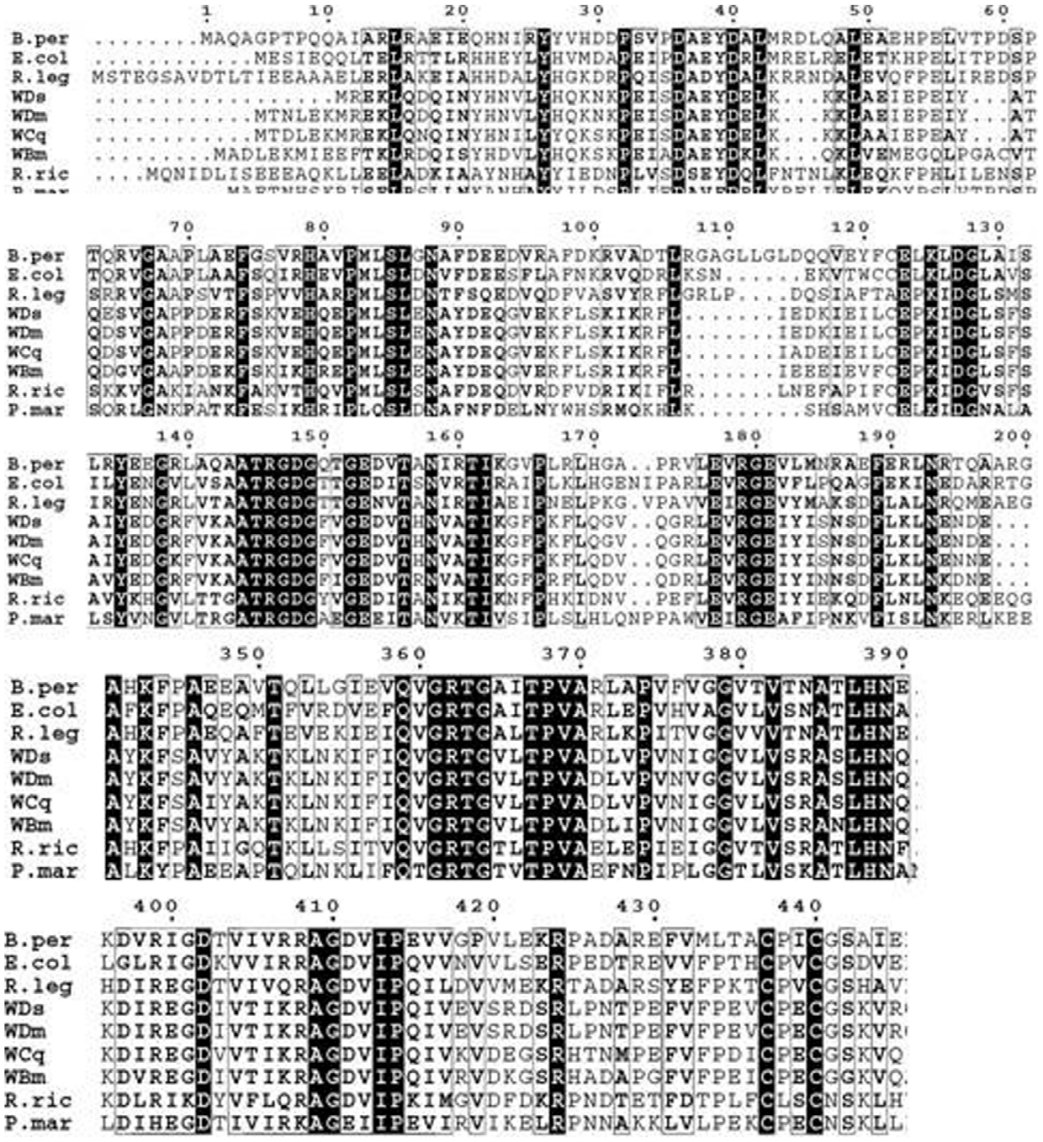
Clustal alignment of amino acid sequence of *w*Bm-LigA with other prokaryotic bacteria. Amino acid sequence of *w*Bm-LigA (GenBank:AAW71136.1) wasaligned with amino acid sequences of DNA ligases of other prokaryotic bacteria including alphaproteobacteria, *Rhizobium leguminosarum* (R.leg, reference sequence YP_002282076.1), *Rickettsia rickettsii* (R.ric, GenBank:ABY73051.1), *Wolbachia* endosymbiont of *Drosophilamelanogaster* (WDm, reference sequence NP_966531.1), *Drosophila simulans* (WDs, reference sequence ZP_00372215.1), *Culex quinquefasciatus* (WCq, reference sequence accession YP_001975247.1); Betaproteobacteria, *Bordetella pertussis*(B.per, reference sequence NP_882073.1.); Gamma proteobacteria, *Escherichia coli* (E.col, GenBank:AAN81395.1**)** and cyanobacteria *Prochlorococcus marinus* (P.mar, reference sequence NP_876232.1). Residues that are identical are highlighted in black, and the conserved amino acid changes are outlined in gray rectangular boxes.

**Figure 3:**
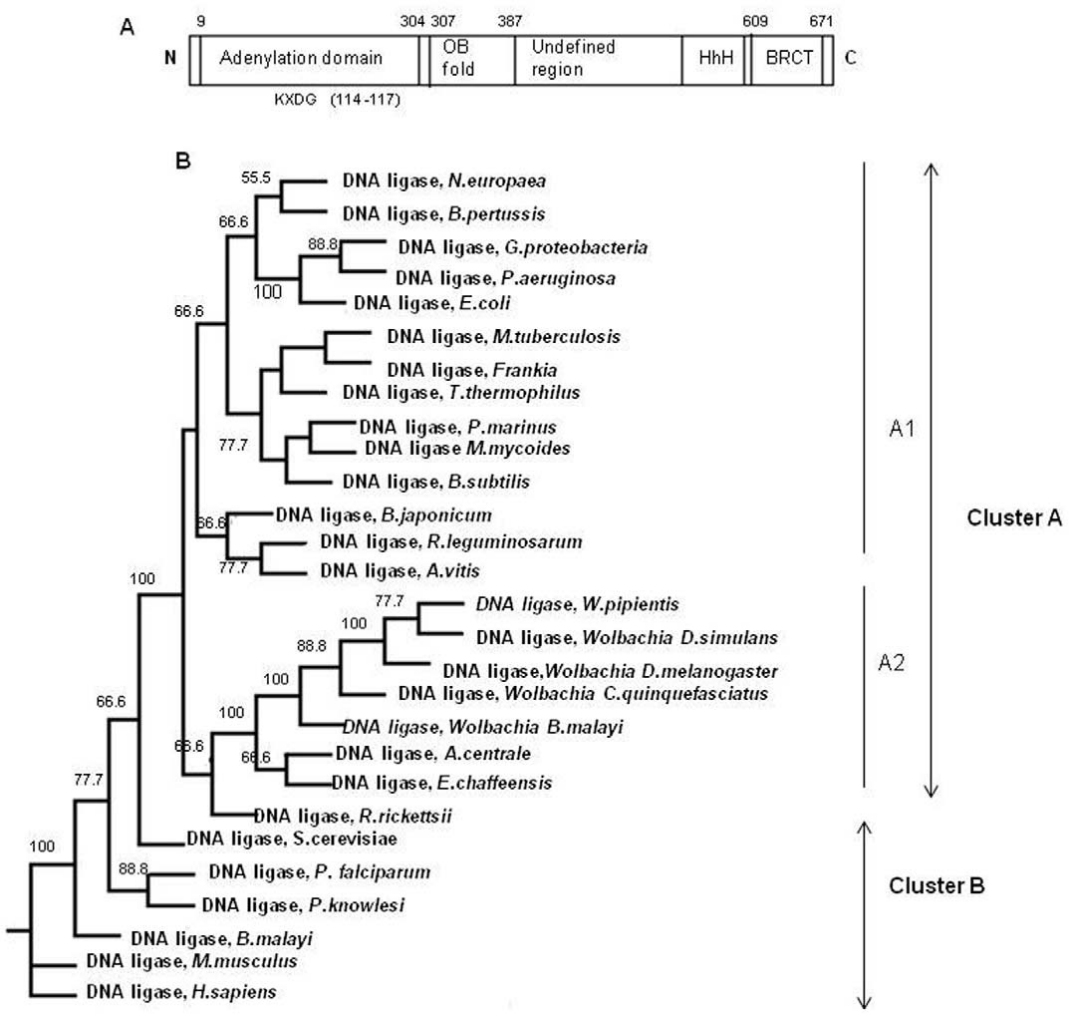
Conserved domain architecture of *w*Bm-LigA and phylogenetic tree of DNA ligases from various organisms. (A) Conserved domain architecture of *w*Bm-LigA typical of DNA ligase superfamily includes adenylation domain (9–304) with KXDG catalytic site(114–117) involved in adenylation reaction, OB fold domain(307–387), HhH (helix hairpin helix) and BRCT domain (609–671). (B) Phylogenetic trees were constructed with Maximum likelihood (ML) methods using PROML programs of PHYLIP 3.69. All the prokaryotic NAD^+^-dependent DNA ligase bacteria formed a discrete cluster A which is divided into two branches, the first one comprising the subcluster A1 constituting *Nitrosomonas europaea* (reference sequence NP_841783.1), *Bordetella pertussis* (reference sequence NP_882073.1), *Gammaproteobacteria* (reference sequence ZP_05126247.1), *Pseudomonas aeruginosa* (reference sequence YP_791724.1), *Escherichia coli* (GenBank:AAN81395.1), *Mycobacterium tuberculosis* (reference sequence ZP_7441467.2), Frankia (reference sequence ZP_06413452.1), *Thermus thermophilus* (GenBank:AAD13190.1), *Prochlorococcus marinus* (reference sequence NP_876232.1*), Mycoplasma mycoides* (reference sequence NP_975740.1), *Bacillus subtilis* (reference sequence NP_388544.1), *Bradyrhizobium japonicum* (GenBank:BAC51856.1), *Rhizobium leguminosarum* (NCBI reference sequence YP_002282076.1), *Agrobacterium vitis* ( reference sequence YP_002550080.1) and the second one (subcluster A2) comprising the DNA ligase of *Wolbachia* endosymbiont present in *Brugia malayi* (GenBank:AAW71136.1) and insects as *Drosophila melanogaster* (reference sequence NP_966531.1), *Drosophila simulans* (reference sequence ZP_00372215.1) and *Culex quinquefasciatus* (reference sequence YP_001975247.1), *Wolbachia pipientis* (GenBank:AAY81980.1), *Anaplasma centrale* (reference sequence YP_003328904.1), *Ehrlichia chaffeensis* (NCBI reference sequence YP_507122.1) and *Rickettsia rickettsii* (GenBank:ABY73051.1). All the eukaryotic DNA ligase viz. *Homo sapiens* (reference sequence NP_000225.1), *Mus musculus* (reference sequence NP_001186239.1), *Brugia malayi* (NCBI reference sequence XP_001896804.1), *Plasmodium falciparum* (GenBank:AAN64156.1), *Plasmodium knowlesi* (reference sequence XP_002261933.1) and *Saccharomyces cerevisiae* (GenBank:CAA91582.1) are present in discrete cluster B indicating *Wolbachia* endosymbiont are distantly related to the ATP DNA ligase of either its host parasite *B. malayi or H.sapiens.*

The Clustal alignment of amino acid sequences of NAD^+^-dependent DNA ligase from an array of prokaryotic species including alpha, beta, gamma proteobacteria and cyanobacteria indicated that the *w*Bm-LigA was 79% identical, 89% similar to DNA ligase of *Wolbachia* endosymbiont of *Drosophila melanogaster*; 79% identical, 90% similar to *Wolbachia* endosymbiont of *D. simulans*; 78% identical, 88% similar to *Wolbachia* endosymbiont of *Culex quinquefasciatus*; 45% identical, 62% similar to DNA ligase of *Rickettsia rickettsii*; 42% identical, 59% similar to *Rhizobium leguminosarum*; 40% identical, 54% similar to *Bordetella pertussis*; 40% identical, 58% similar to *E. coli* and 37% identical, 55% similar to *Prochlorococcus marinus* (Figure 2). The conserved domain architecture, unique to DNA ligase superfamily, was identified in *w*Bm-LigA. Residue 9 to 304 constitute an adenylation domain (LIGANc) carrying KXDG catalytic motif (114–117amino acid), involved in adenylation reaction. C-terminal region has an OB fold domain (amino acid 307–387), HhH (helix hairpin helix motif) and BRCT domain (amino acid 609–671), all characteristics of the NAD^+^-dependent DNA ligase superfamily (Figure 3A). Analysis of the phylogenetic tree (Figure 3B) showed that *w*Bm-LigA forms a discrete cluster A with closely related NAD^+^-dependent DNA ligase of all the prokaryotic bacteria which is subdivided into two branches, the first one constituting the subcluster A1 comprised of *Nitrosomonas europaea, Bordetella pertussis, Gamma proteobacteria, Pseudomonas aeruginosa, E. coli, M. tuberculosis, Frankia, Thermus thermophilus, Prochlorococcus marinus, Mycoplasma mycoides, Bacillus subtilis, Bradyrhizobium japonicum, Rhizobium leguminosarum, Agrobacterium vitis* and the second one (subcluster A2) comprising *w*Bm-LigA along with the DNA ligase of *Wolbachia* endosymbiont present in the insects such as *Drosophila melanogaster, D. simulans*, *Culex quinquefasciatus,* other alpha proteobacteria such as *Wolbachia pipientis, Anaplasma centrale, Ehrlichia chaffeensis and Rickettsia rickettsii.* All the eukaryotic DNA ligases which are ATP dependent viz. *Homo sapiens, Mus musculus, B. malayi, Plasmodium falciparum, P. knowlesi and Saccharomyces cerevisiae* are present in a separate cluster B indicating significant divergence between *w*Bm-LigA and *B. malayi* or *H. sapiens*.

### Structural analysis of recombinant *w*Bm-ligA, circular dichroism and fluorescence spectra

**Figure 4:**
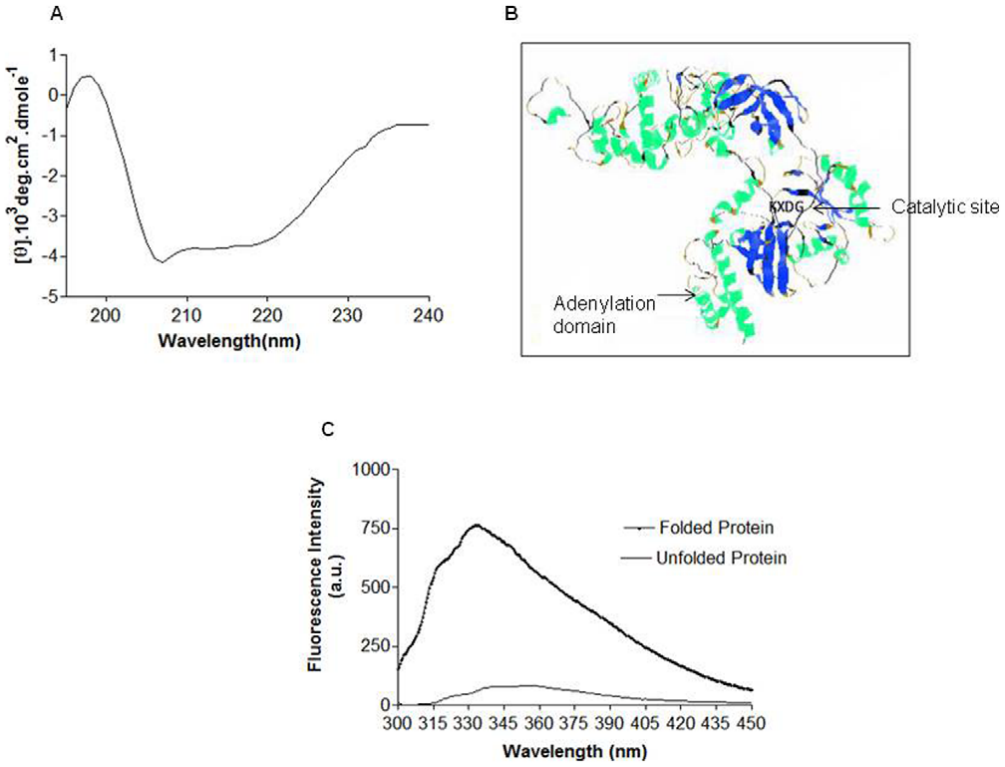
CD Spectra, 3D-JIGSAW model and fluorescence spectra. (A) CD spectra of recombinant *w*Bm-LigA, recorded in far UV region. CD spectra gave 2 negative bands at 207 nm and 220 nm, and slight positive ellipticity at 196nm. (B) Ribbon diagram of 3D structure model of *w*Bm-LigA made by 3D-JIGSAW and visualized by Rasmol. α Helices is represented in green; β strand in blue and turns in brown. Model indicated a total number of 26 α-helices and 29 β-strands including 3 β sheets and 69 turns. Conserved active site residues KXDG (amino acid residues 114–117) are also indicated. (C) Fluorescence emission spectrum of *w*Bm-LigA recorded between 300 nm and 450 nm wavelength after excitation at a 280 nm. Maximum peak was generated at 333 nm indicating folded form of *w*Bm-LigA that was denatured by Gu-HCl resulting in red shift in spectra and decrease in the fluorescence intensity.

The circular dichroism (CD) spectra revealed 2 negative bands at 207 nm and 220 nm, and marginally positive ellipticity at 196 nm (Figure 4A). K2D2 web server was employed to estimate the ratio of secondary structure elements. K2D2 server accepts a CD spectrum as an input and gives out the estimated secondary structure content of the corresponding protein as an output. The deconvolution of the CD spectra with K2D2 indicated a secondary structure ratio of 36.44% α-helix, 12.07% β strands that were consistent with the predicted values (GOR1V method) of 44.51% α-helix and 14.20% β strands. The 3D structure of *w*Bm-LigA was deduced from 3D-JIGSAW automated web server and is represented as a ribbon diagram in Figure 4B. This model indicates a total of 26 α-helices and 29 β strands including 3 β sheets and 69 turns. The conserved active site residues KXDG (amino acid residues 114–117) are also displayed in the figure. The fluorescence emission spectrum of the purified *w*Bm-LigA protein in standard buffer at 25°C showed maximum emission at 333 nm when excited at 280 nm. Guanidium hydrochloride induced decrease in the intrinsic fluorescence intensity and a red-shift in the emission maximum wavelength, indicating denaturation of the protein (Figure4C).

### *w*Bm-LigA demonstrates the nick closing activity

**Figure 5:**
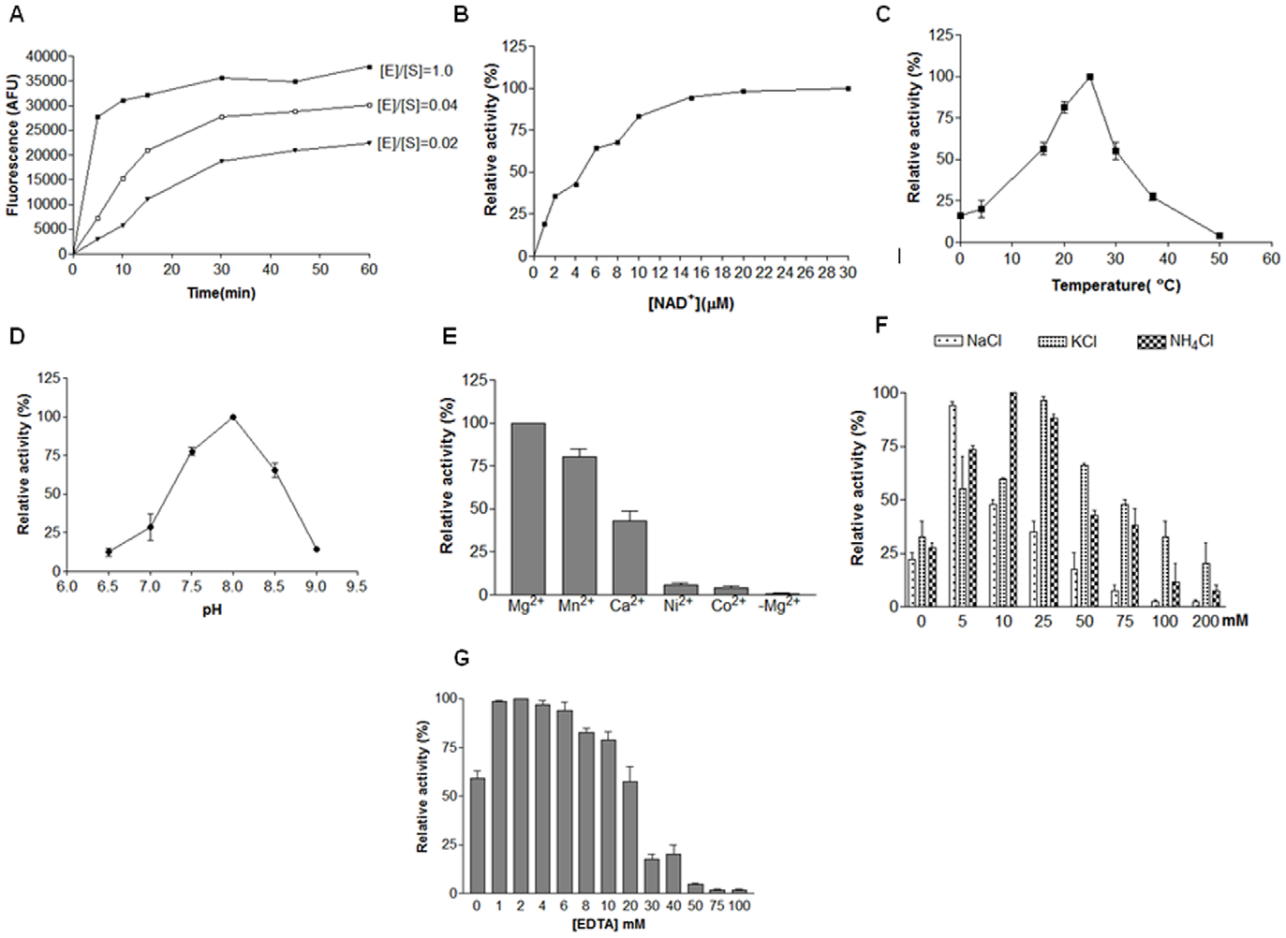
Nick closing activity of *w*Bm-LigA and biochemical characterization. (A) Effect of enzyme substrate ratio on nick closing activity. Incubation of an equimolar ratio of enzyme and substrate caused rapid product formation and quick saturation (close squares). Decreasing the enzyme/substrate ratio to 0.04 or 0.02 produced slower product formation (open squares and triangles res.). (B) Effect of varying concentration of NAD^+^ cofactor. Increasing concentrations of NAD^+^ showed a rise in *w*Bm-LigA activity which plateaued at about 10 µM. (C) Effect of temperature on enzymatic activity ranging from 0°C –50°C. Enzyme showed maximum activity at 25°C. (D) Nick closing activity at pH ranging between 6.5 and 9.0 with highest activity at 8.0. (E) Effect of divalent cations on enzyme activity. Enzyme was active in presence of 10 mM MgCl_2_, MnCl_2_ and CaCl_2_. However, MnCl_2_ and CaCl_2_ showed less product formation as compared to the MgCl_2_. Enzyme showed no activity in presence of NiCl_2_ and CoCl_2._ (F) Effect of different concentrations of monovalent cations including NaCl, KCl and NH_4_Cl. Maximal activity achieved at 5 mM NaCl which was completely inhibited at 100 mM. In presence of KCl, maximum activity was obtained at 25 mM showing a sharp decline thereafter. Activity increases with increasing concentration of NH_4_Cl up to 10 mM, beyond this the enzymatic activity declined. (G) Effect of varying concentrations of EDTA on the ligation activity of *w*Bm-LigA. Error bars represent standard errors calculated from two independent experiments.

A concentration-response curve of *w*Bm-LigA nick activity was generated to show the proportionality of the enzyme concentration to the nicked substrate. Incubation of an equimolar ratio of enzyme and substrate caused rapid product formation and quick saturation. Decreasing the enzyme/substrate ratio to 0.04 or 0.02, produced slower product formation (Figure 5A). Using varying concentration of the nicked substrate, a Km of 0.193 µM was obtained for *w*Bm-LigA. The very first step in ligation reaction is the adenylation of the ligase enzyme requiring AMP moiety that is provided by the NAD^+^ cofactor. The increasing concentrations of NAD^+^ showed a rise in *w*Bm-LigA activity which plateaued at about 10 µM concentration (Figure 5B). The Km of 4.8 µM for NAD^+^ was obtained for *w*Bm-LigA. NAD^+^-dependent DNA ligase had an optimum activity at 25°C (Figure 5C). Optimal pH range was 7.5–8.5 with pH maxima at 8.0 for the nick closing activity (Figure 5D) and more than 60% activity was retained within this pH range. As with the other known NAD^+^-dependent DNA ligases, *w*Bm-LigA activity was also very sensitive to ionic strength. The monovalent as well as divalent ions are required for the enzymatic activity. The divalent cations needed for catalysis can be fulfilled by some of the cations apart from magnesium. The maximum enzymatic activity was obtained in the presence of 10 mM MgCl_2_. However, MnCl_2_ and CaCl_2_ also suffice for this requirement. In contrast, Ni^2+^ and Co^2+^ hardly affected the ligation reaction (Figure 5E). The enzyme activity increased in the presence of a monovalent ion such as NaCl, KCl and NH_4_Cl. Maximal activity occurred at 5 mM NaCl, however, further increase in the concentration completely inhibited this activity viz. no activity could be observed at 100 mM NaCl. In presence of KCl, the maximum activity was achieved at 25 mM concentration showing a sharp decline thereafter. The activity increased with the increasing concentrations of NH_4_Cl up to 10 mM, beyond that the activity decreased (Figure 5F). The enzyme activity increased in the presence of low concentrations of EDTA, however at higher concentration beyond 30 mM, the DNA ligase activity was inhibited. (Figure 5G).

### *w*Bm-LigA demonstrates the cohesive end ligation activity

**Figure 6:**
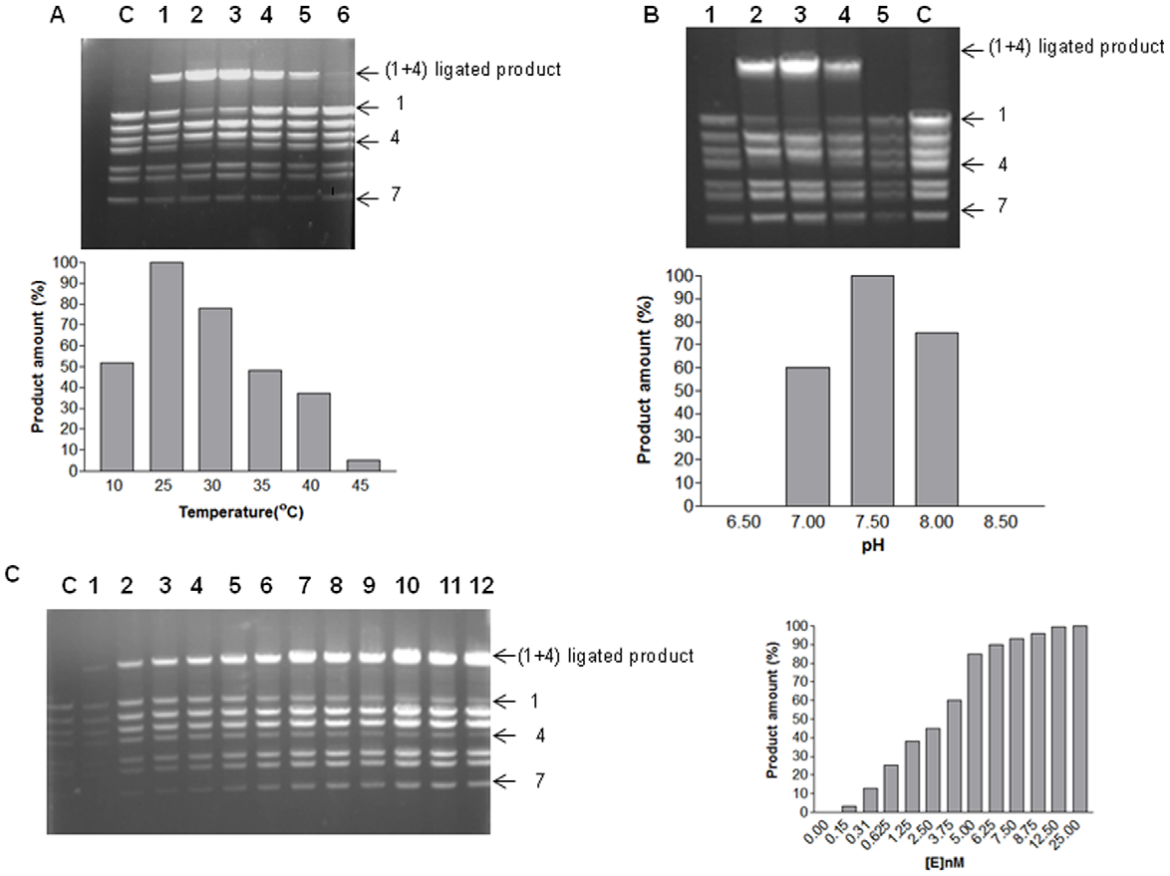
Cohesive end ligation activity of *w*Bm-DNA ligase at various temperature, pH and enzyme concentration. (A) Temperature dependence of cohesive end ligation activity of *w*Bm-LigA. Lane 1, 2, 3, 4, 5, 6 showed ligation at 10°C, 25°C, 30°C, 35°C, 40°C and 45°C temperature res.; lane C, control in which no enzyme was added. Enzyme showed maximum ligation at 25°C. Quantitation of the data is shown below as a bar diagram. (B) pH dependence on the cohesive end ligation. Lane 1, 2, 3, 4, 5 represents ligation at 6.5, 7.0, 7.5, 8.0, 8.5 pH res.; lane C, control. Maximum ligation activity was observed at 7.5 pH. Quantitation of the data is shown below as a bar diagram. (C) (Lanes 1–12), effect of enzyme concentration (0–25nM) on cohesive end ligation; Lane C, control. Quantitation of the data is shown to the right as a bar diagram. Ligation of cohesive ends of fragment 1 and fragment 4 of lamda DNA digested with BstEII into 14 kb ligated product was analyzed on a 1.0% agarose gel. Activity values were calculated by measuring the net intensity of the product band (indicated as 1+4 in the figures) relative to a non-substrate fragment (fragment 7).

**Figure 7:**
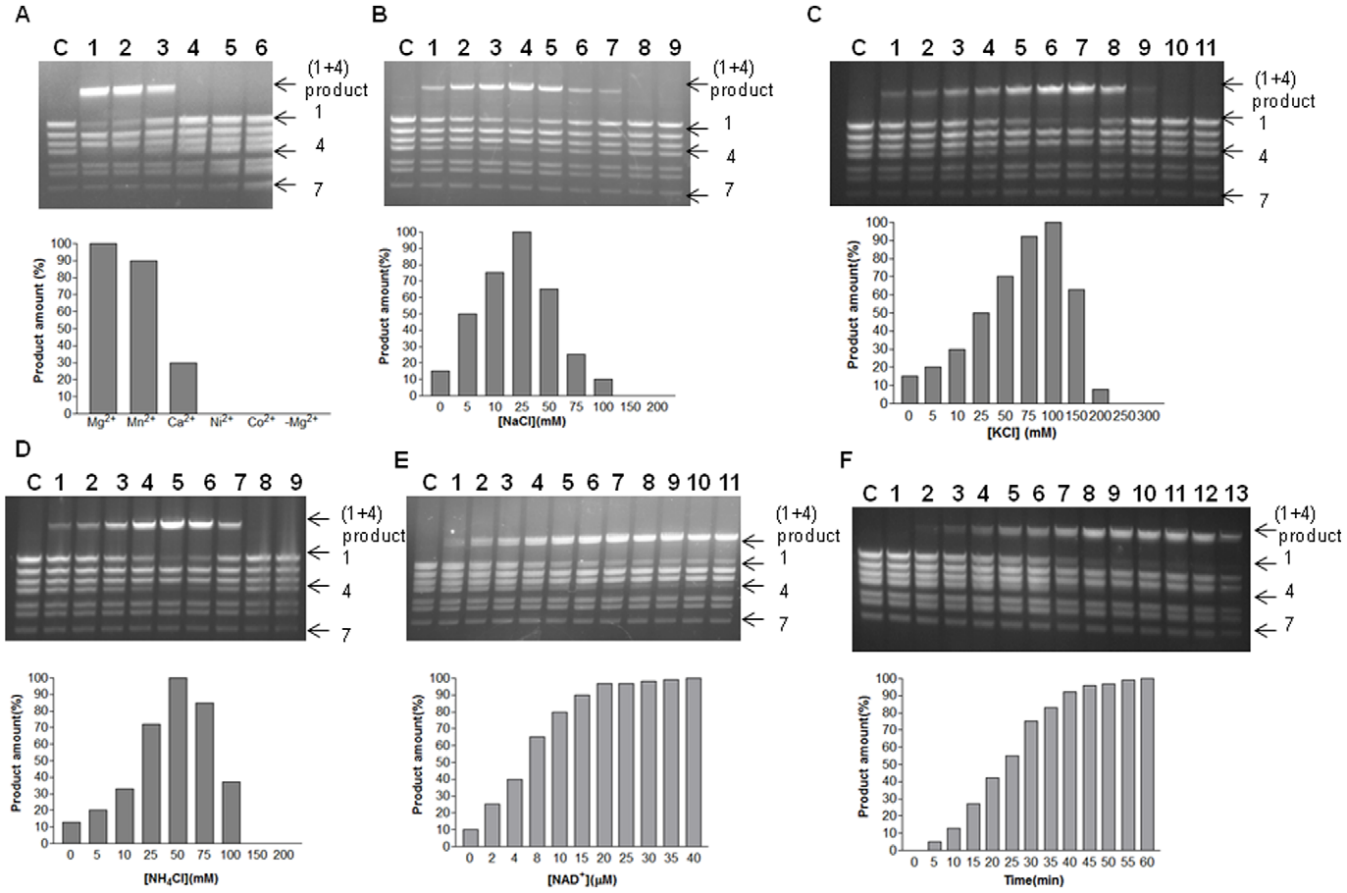
Effect of salts, NAD^+^ cofactor on cohesive end ligation activity and time course of ligation. (A) Cohesive end ligation activity in presence of, lane 1, 10 mM MgCl_2_; lane 2, 10 mM MnCl_2_; lane3, 10 mM CaCl_2_; lane 4, 10 mM NiCl_2;_ lane 5, 10 mM CoCl_2_; lane 6, no MgCl_2_; lane C, control with no added enzyme. (B) (Lanes 1–9), DNA ligase activity assays performed at increasing NaCl concentrations from 0 to 200 mM; lane C, control with no added enzyme. (C) (Lanes 1–11), DNA ligase activity assays performed at increasing KCl concentrations from 0 to 300 mM; lane C, control with no added enzyme. (D) (Lanes 1–9), DNA ligase activity assays performed at increasing NH_4_Cl concentrations from 0 to 200 mM; lane C, control with no added enzyme. (E) (Lanes 1–11), effect of varying concentrations of NAD^+^ cofactor; lane C, control with no added enzyme. (F) (Lanes 1–13), time course of ligation of the cohesive ends at different time interval starting from 0 min to 60 min; lane C, control in which no enzyme was added. Quantitation of the data is shown below each gel image as a bar diagram.

*w*Bm-LigA was able to catalyze the ligation of cohesive ends of lamda DNA digested with BstEII enzyme. Optimum temperature for cohesive end ligation was 25°C similar to the nick closing activity (Figure 6A). Optimal pH range was 7.0–8.0 with maximum activity at pH 7.5 (Figure 6B). With increasing enzyme concentration, the product formation also increased initially and then it became constant (Figure 6C). The monovalent as well as divalent cations were required for the cohesive end ligation activity. The maximum enzymatic activity was obtained in the presence of 10 mM MgCl_2_ whereas 10 mM concentrations of MnCl_2_ and CaCl_2_ resulted into 90% and 30% product formation respectively. The ligation activity was not observed in presence of Ni^2+^ and Co^2+^ (Figure 7A). The ligation activity was maximum in the presence of 25mM NaCl, 100 mM KCl or 50 mM NH_4_Cl (Figures 7B, 7C and 7D). The increasing concentrations of NAD^+^ showed a rise in *w*Bm-LigA cohesive end ligation activity up to 20 µM concentration (Figure 7E). Time course of ligation of BstEII digested lamda DNA fragments into the product revealed that product quantity was maximum by 45 min and longer incubations periods did not result in an increase in product amount (Figure 7F).

### *w*Bm-LigA is present in all the major life-cycle stages of *B. malayi*

**Figure 8:**
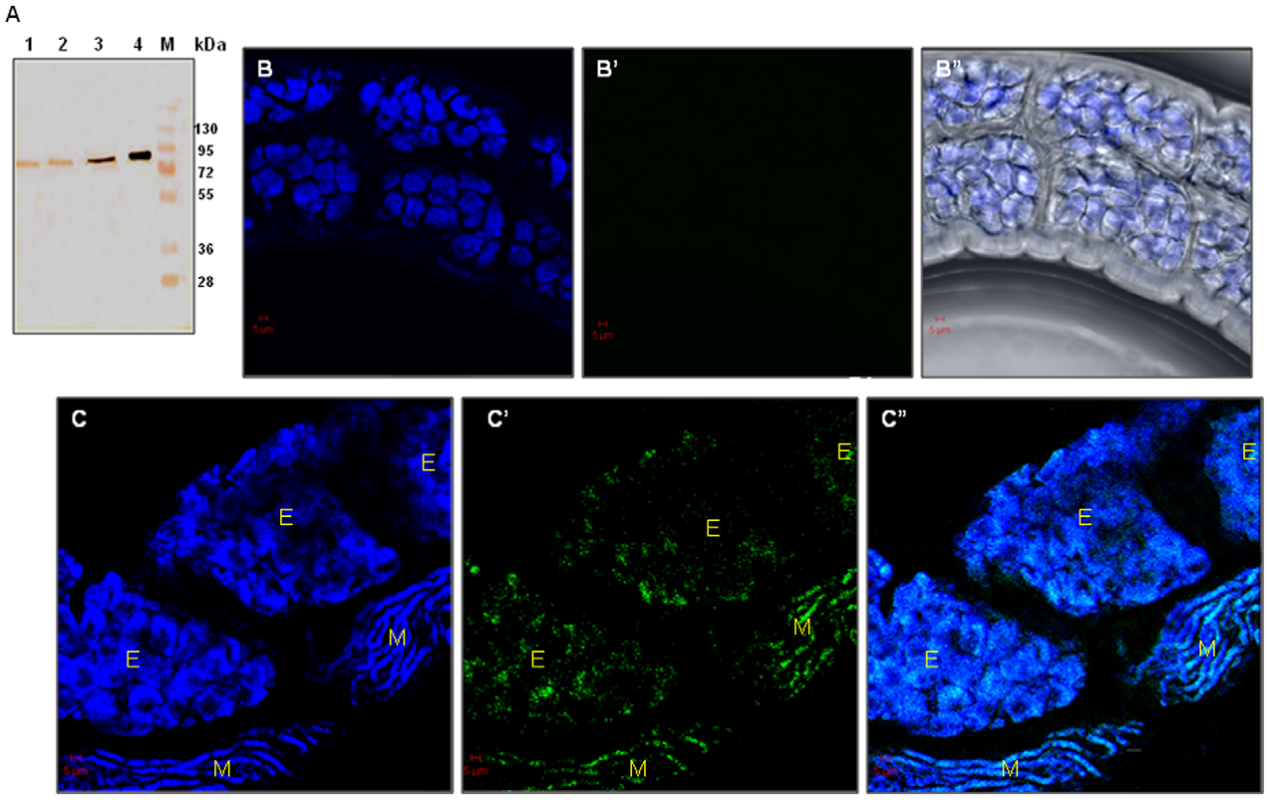
Stage-specific expression of *w*Bm-LigA by immunoblotting and immunolocalisation in female adult worm by confocal microscopy. (A) The stage-specific expression of *w*Bm-LigA. Western blot was done with anti-*w*Bm-LigA antibody to confirm presence/absence of *w*Bm-LigA. Lane 1, microfilariae; lane 2, infective larvae; lane 3, adult worms (both sexes) of *B. malayi*; lane 4, purified *w*Bm-LigA (positive control); lane M, standard protein molecular weight marker. *w*Bm-LigA was detected in all the three major life stages. (B–B″, C–C″) Immunolocalisation of *w*Bm-LigA in *B.malayi* female adult worm. (B–B″) Adult worm treated with pre-immune serum as primary antibody followed by fluorescein isothiocyanate (FITC, green) conjugated secondary antibody and counterstained with 4′,6′-diamidino-2-phenylindole (DAPI, blue). (B) Fluorescence signal generated with DAPI. (B′) No green signal in the control adult worm. (B″) Phase contrast image. (C–C″) Female adult worm incubated with mouse anti-*w*Bm-LigA serum followed with FITC conjugated secondary IgG and counterstained with DAPI. (C) Fluorescence signal generated with DAPI. (C′) Fluorescence signal generated by FITC conjugated secondary IgG indicating the distribution of *w*Bm-LigA in embryos (E) and stretched microfilariae (M) in female adult worm. (C″) Merge image of the fluorescence emitted by DAPI and FITC in C and C′. All the images were captured at 40X objective. Scale Bar: 5μm.

*w*Bm-LigA antiserum generated in BALB/c mice reacted in the blot with endogenous *w*Bm-LigA in the crude extracts prepared from the three major life-stages of *B. malayi* viz. infective larve (L3), adult worms and mf, showing a characteristic band at ∼73 kDa. The purified recombinant *w*Bm-LigA which acted as positive control also showed reaction with anti-*w*Bm-LigA antibody giving a characteristic band at ∼75 kDa including His-tag at C and N terminus (Figure 8A).

### The confocal microscopy localized the endogenous *w*Bm-LigA in adult *B. malayi* and mf

**Figure 9:**
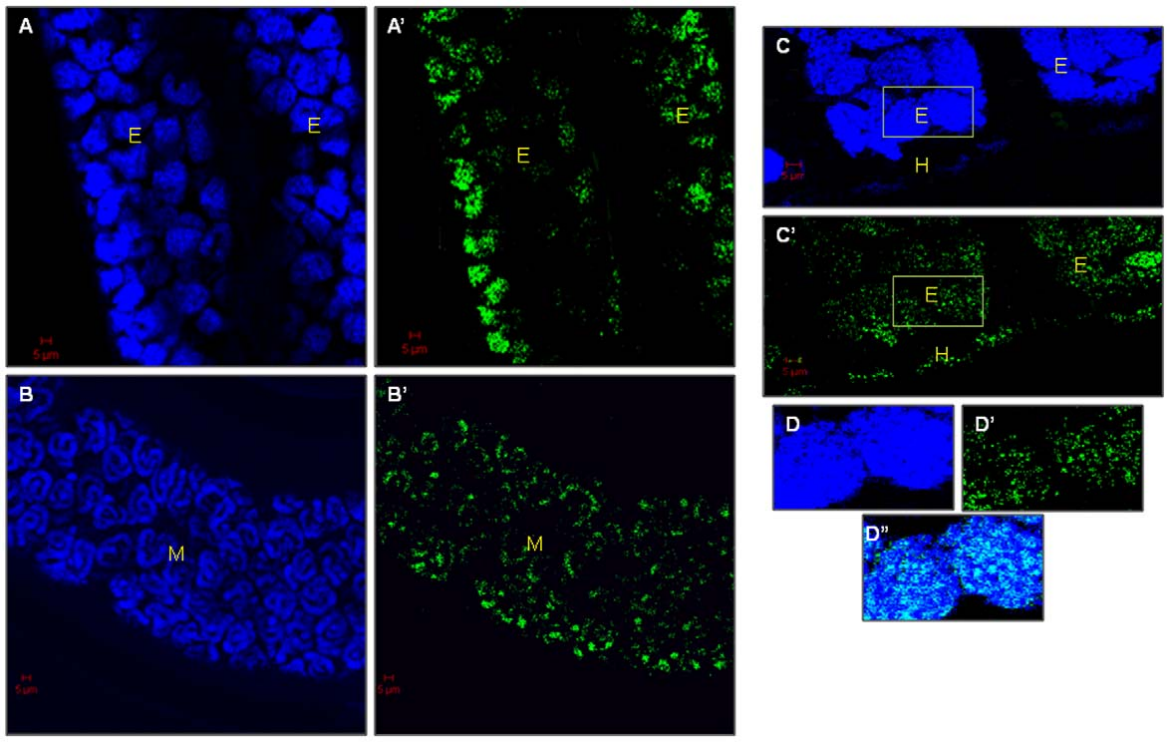
*w*Bm-LigA presence in the developing embryos, mf and hypodermal chords of female adult worm. (A, A′) Adult female worm images through embryonic region. (A) Fluorescence signal generated by DAPI. (A′) Fluorescence signal generated by FITC conjugated secondary IgG indicating the presence of *w*Bm-LigA in the developing embryos. (B, B′) Adult female worm images showing intrauterine mf. (B) Fluorescence signal generated with DAPI. (B′) Fluorescence signal generated by FITC conjugated secondary IgG indicating the distribution of *w*Bm-LigA in intrauterine mf (M). (C, C′) Adult female worm images showing developing embryos and hypodermal chords. (C) Fluorescence emitted by DAPI. (C′) FITC signal indicating the presence of *w*Bm-LigA in hypodermal chords (H) and embryos (E). (D, D′) Magnification of square in (C, C′). (D″) Merge image of D and D′. A and A′ images were captured at 40X objective and other images were at 63X oil objective. Scale Bar: 5μm.

**Figure 10:**
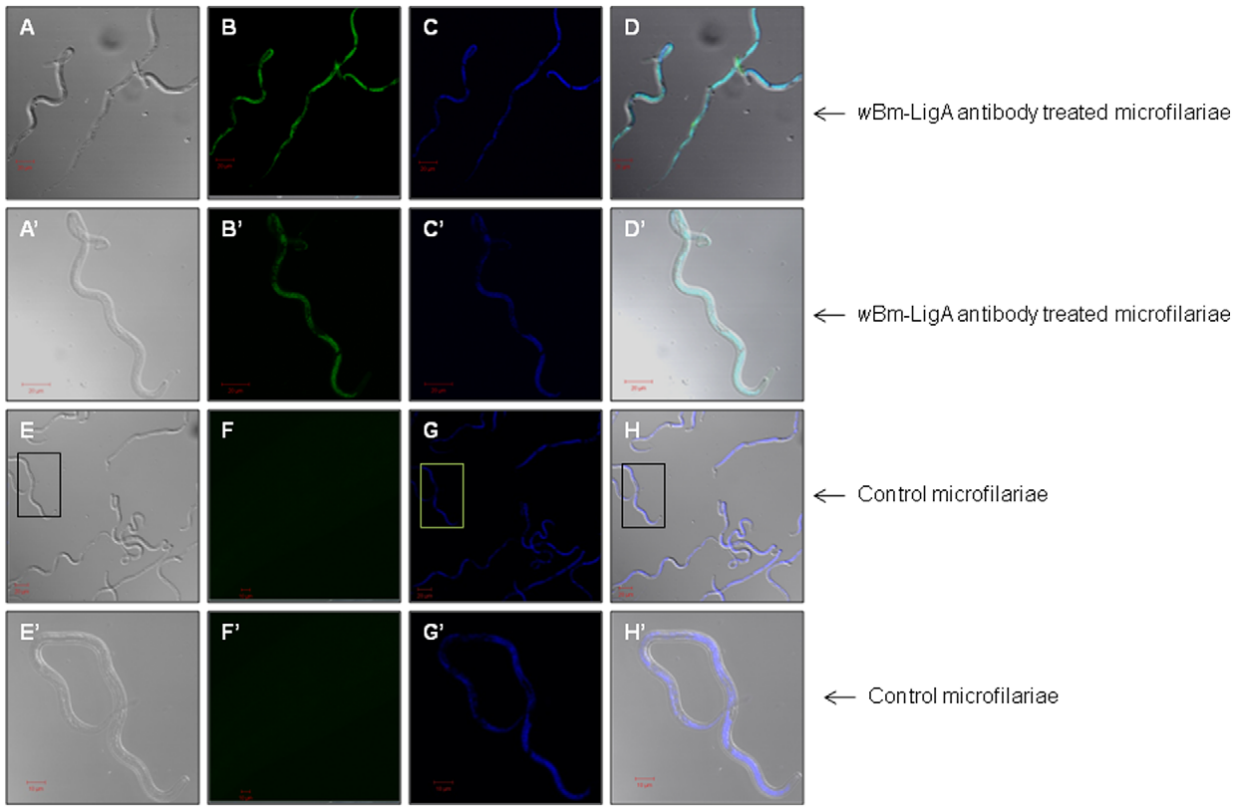
Immunolocalisation of *w*Bm-LigA in *Brugia malayi* microfilariae by confocal microscopy. (A–D, A′–D′) Microfilariae were incubated with anti-*w*Bm-LigA antibody followed by incubation with FITC conjugated secondary antibody and counterstaining with DAPI. (A, A′) Phase contrast image of microfilariae. (B, B′) Green fluorescence signal generated by FITC indicating distribution of *w*Bm-LigA. (C, C′) Blue signal produced by DAPI indicating presence of nuclear DNA. (D, D′) Merge image of the phase contrast and fluorescence emitted by DAPI and FITC. (A′–D′) are images of single microfilariae at higher magnification (40X). (E–H, E′–H′) Microfilariae incubated with BALB/c preimmune serum followed by incubation with FITC conjugated secondary antibody and counterstaining with DAPI served as control. (E, E′) Phase contrast image. (F, F′) No green fluorescence signal of FITC. (G, G′) Blue signal generated with DAPI. (H, H′) Merge image of the phase contrast and fluorescence emitted by DAPI. (E′–H′) are images of single microfilariae indicated in boxes in (E–H res.) at higher magnification (40X).

The polyclonal antibody raised in BALB/c mice against *w*Bm-LigA reacted with the endogenous *w*Bm-LigA protein inside the adult female parasite and mf in confocal microscopy. In the case of adult female worm, high level of *w*Bm-LigA expression could be detected inside the uteri in the developing embryos, stretched mf as well as hypodermal lateral chords indicated by green fluorescence (fluorescein isothiocyanate, FITC) (Figures 8C′; 9A′, 9B′ and 9C′). In contrast, adult worm treated with preimmune BALB/c serum did not reveal any green signal for *w*Bm-LigA presence in adult worms (Figure 8B′). 4′,6′-diamidino-2-phenylindole (DAPI, blue) stained *Wolbachia* and *B. malayi* DNA (Figures 8B, 8C; 9A, 9B and 9C). *w*Bm-LigA was also conspicuous as green fluorescence in isolated mf (Figure 10B and 10B′) in contrast to mf, treated with preimmune serum (Figure 10F and 10F′).

## Discussion

The wolbachial growth and multiplication is essential for the survival and fertility of its host, *B. malayi*. DNA ligases are essential actors in DNA replication by virtue of their ability to join the Okazaki fragments together to form a continuous lagging strand. Therefore, DNA ligase of *Wolbachia* plays an indispensable role in the intracellular bacterial division inside the *B. malayi* parasite. It is also involved in the maintenance of genetic stability during DNA damage and in bacterial recombination. The characterization of *w*Bm-LigA is therefore an important step towards understanding the reactions involved in DNA repair and replication at molecular level in this obligate mutualist (*w*Bm) and conclusion drawn from the following study would further assist in validating its drug target potential.

The indispensable nature of this enzyme is attributed to the fact that it is highly conserved across the different bacterial species as evident by the protein sequence alignment that indicated considerable sequence similarity between *w*Bm-LigA and other bacterial DNA ligases including alpha, beta, gamma bacteria as well as blue-green bacteria. DNA ligases of alphaproteobacteria formed a distinct clade in phylogenetic tree which was also strongly supported by bootstrap scores, leads us to speculate that DNA ligase of alpha proteobacteria are evolutionary closely related to each other and they may have followed a different evolutionary path from rest of other bacteria. The possible reason could be the lifestyles in which alphaproteobacteria have to survive and divide. It is already well established that the alphaproteobacteria constitute an excellent model system to explore how the bacterial genomes have evolved and how the genomic features are related to environmental adaptation [17]. Earlier studies also demonstrated that α-proteobacteria can be clearly distinguished from all other bacteria based upon a large number of molecular characteristic and therefore should be recognized as a main group or phylum within bacteria, rather than as a subdivision or class of the proteobacteria [18]. A significant divergence of *w*Bm-LigA from eukaryotic DNA ligases of *H. sapiens, M. musculus, B. malayi, S. cerevisae* and *Plasmodium* species which are ATP dependent, indicates the potential of *w*Bm-LigA as an attractive antifilarial drug target.

*w*Bm-LigA has a modular structure typical of NAD^+^-dependent DNA ligases and each step of ligation pathway depends upon a different subset of these modules. However, tetracysteine zinc finger domain which is important in the formation of stable complex with DNA substrate in ligation reaction is not present in *w*Bm-LigA. This feature is in contrast to that of *R. rickettsii* as well as *E. coli* DNA ligase which possess C4 type Zn domain for DNA binding. It is possible that being an endosymbiont in nature, *Wolbachia* has retained only the essential domains and deleted those, whose function could be provided in trans by some other factors conceivably by undefined region of *w*Bm-LigA or might be HhH motif and BRCT domain could be solely responsible for stable DNA binding. The conserved KXDG catalytic site in the N terminal domain of *w*Bm-LigA is formed by a single β strand and the random coils while the C-terminal region of *w*Bm-LigA is predominantly composed of the alpha helices as evident by the homology model. Published reports implicate C-terminal domain of NAD^+^-dependent DNA ligase in recognition of DNA substrate principally through the protein-protein interaction [19], [20]. Besides, the BRCT domain also acts as a signal transducer transmitting the signal from DNA damage sensors to the other components of the DNA damage-responsive checkpoint machinery via specific protein–protein interaction in DNA repair system [21]. Thus, homology model structure clearly confirms that C-terminal region acts through protein-protein interaction since alpha helices are known to play a prime role in protein-protein interaction as well as in DNA binding. Thus, the presence of all the characteristic domains of DNA ligase superfamily in DNA ligase of endosymbiont *Wolbachia* demonstrates the remarkable ability of *Wolbachia* for self perpetuance in its host.

Immunoblotting studies with crude extract of adult worm, L3 and mf indicated the presence of *w*Bm-LigA in all the major life-stages of *B. malayi*. *In vivo* localization of *w*Bm-LigA by immunoconfocal microscopy using anti-*w*Bm-LigA antibody demonstrated the presence of enzyme in the hypodermal chord of female adult worm as well as inside the uteri of female parasite i.e. in the developing embryos and mf. The enzyme showed its presence also in isolated mf. The ligA gene expression in all the major life-stages of *B. malayi* further indicated the essentiality of this enzyme for the parasite. In several organisms, DNA ligases have been reported to be essential [13], [15], [16], and therefore there is great likelihood that it is also essential in *Wolbachia* thereby suggesting its significance as an antifilarial drug target.

The full-length *w*Bm-LigA expressed as the recombinant protein in *E. coli* was enzymatically active when quantitated in an *in vitro* ligation assay using a fluorescent labeled substrate in nick closing activity assay and BstEII digested lamda DNA as a substrate in the cohesive end ligation assay. The biochemical characterization established that the *w*Bm-LigA requires an optimal temperature 25°C, optimal pH range 7.5–8.5 for nick closing activity and pH 7.0–8.0 for cohesive end ligation activity displaying its strict pH dependent nature. The data reveal that *w*Bm-LigA fulfills the requirement of a divalent metal cofactor by magnesium cations which are most often associated with ligation activity. It is worth mentioning that *w*Bm-LigA was able to catalyze nick sealing as well as cohesive end ligation even in the presence of Mn^2+^ as well Ca^2+^, since Ca^2+^ has a larger ionic radius than Mg^2+^ or Mn^2+^ this suggests more flexibility within the “catalytic-core” of *w*Bm-LigA which is evident from 3D homology model where a large portion of catalytic site is formed by the random coils. *w*Bm-LigA possesses high affinity for the nicked DNA substrate as reflected by its lower Km (0.193µM) than that of *E. coli* DNA ligase (0.702µM) [22]. Lower Km value for the NAD^+^ cofactor could be attributed to the fact that *w*Bm lacks complete pathways for de novo biosynthesis of cofactor NAD^+^ which makes *w*Bm-LigA, dependent on the host for the supply of the precursors [3]. Reactions carried out at increasing concentrations of EDTA and fixed concentration of Mg^2+^ indicated increased enzymatic activity with concentrations below 20 mM of EDTA. The reason could be that at these concentrations of EDTA, the Mg^2+^ that is available to *w*Bm-LigA is optimal for its activity. The other point is that enzymatic activity remain high even when EDTA is at equimolar concentration of Mg^2+^. This could be hypothesized that EDTA although chelates Mg^2+^, but due to high affinity of *w*Bm-LigA for Mg^2+^, available Mg^2+^ to *w*Bm-LigA is sufficient for the increased activity. However, EDTA at 30 mM or higher concentration could efficiently compete and strip the Mg^2+^ resulting into inhibition of *w*Bm-LigA activity. There are reports where small amount of EDTA are being used in the ligation reaction of DNA ligases [13], [23], [24] but their effect on ligation activity is not demonstrated. However, inhibition of DNA ligase by employing high concentration of EDTA (2 or more times than the concentration of the Mg^2+^) is reported for many DNA ligases [25]–[27].

The CD spectra of *w*Bm- LigA in far-UV range showed the presence of a typical α/β type secondary structure. The alpha helix gives negative band at 208 and 222 nm, while the presence of β sheet gives negative band at 218 nm causing a shift in the wavelength at 222 nm of the helix structure. This was further confirmed through the CD spectra deconvolution with K2D2. K2D2 is a webserver that estimates the secondary structure content of a given protein from its CD spectra. The fluorescence spectra clearly indicated that the purified recombinant enzyme was properly folded and was present in its native form. A peak at 333 nm which is red shifted after adding guanidine HCl demonstrated that the three tryptophan residues were buried in hydrophobic environment of the protein and become exposed to more polar environment once the native 3D structure is disrupted via denaturation that led to the decreased intrinsic fluorescence intensity [28].

Ours is the first report on the molecular characterization of NAD^+^-dependent DNA ligase of *Wolbachia* which is essential for DNA replication, repair and recombination. The presence of this enzyme in all the major life-cycle stages of *B. malayi,* high level of *in vivo* endogenous expression and conserved domain organization indicated its imperative role in the biology of filarial endosymbiont. Further, profound divergence from the eukaryotic DNA ligases was also found. Since *Wolbachia* play an important role in the development, viability, fertility of filarial nematodes, and they are also responsible for filarial pathology, *w*Bm-LigA presents an attractive antifilarial drug target. The specific inhibitors of this enzyme are known and new chemical structures may be synthesized which will help in validating *w*Bm-LigA as a candidate drug target. The expression and characterization of this protein represents a critical step towards understanding the molecular trappings underlying *Wolbachia* maintenance in filarial parasite thereby providing more insight into mutualistic interaction between *Wolbachia* and its host, *B. malayi.*

## Materials and Methods

### Ethics statement

The animals used in the study were maintained at Laboratory Animal Division of Central Drug Research Institute (CDRI), Lucknow, under pathogen-free conditions. All the animals, experimental procedures of animal use were duly approved by the Institutional Animal Ethics Committee (IAEC).

### Cloning of full length NAD^+^-dependent DNA ligase gene

The full length open reading frame of *w*Bm-ligA gene (2052 bp long) was amplified from the genomic DNA isolated from *B. malayi* adult worms [29] by PCR using gene specific forward primer 5′-GAATTCATGGCTGATTTGGAAAAGATG-3′ and reverse primer 5′-AAGCTTTTCGGACATTCCGAATTTAT-3′. The forward primer contained EcoRI site (underlined) and the reverse primer contained HindIII site (underlined). The amplification of ligA gene was carried out using 1 µM of each primers (Sigma, USA), 200 µM of each dNTPs (Invitrogen, USA), 0.5 unit Taq DNA polymerase (Invitrogen, USA), 1xPCR buffer (Invitrogen, USA), and 1.5 µM of MgCl_2_ (Invitrogen, USA) under the conditions of initial denaturation at 94°C for 10 min, followed by 29 cycles at 94°C for 45 sec, 65°C for 1 min, 72°C for 2 min, and 1 cycle at 72°C for 10 min. The adult worms were obtained from the peritoneal cavity of jird (*Merions unguiculatus*) infected 3–4 months earlier by inoculation of 150–200 L3 intraperitoneally. The amplified gene product was cloned in pTZ57R/T vector as per manufacturer's instructions (Invitrogen, USA) and transformed into competent DH5α *E. coli* cells. The gene constructs were screened for the presence of recombinant plasmid carrying *w*Bm-ligA insert by gene-specific PCR, followed by double digestion of the recombinant plasmid with EcoRI and HindIII (Fermentas, USA) for subcloning in pET 28a(+) vector. The plasmid construct in pET28a(+) was confirmed by further restriction digestion.

### Overexpression, purification and localization of the recombinant protein (*w*Bm-LigA) in Western blot

The construct of pET28a(+)-*w*Bm-ligA was transformed into various *E. coli* strains viz. BL21(DE3), BL21(DE3)pLysS, Rosetta and C41 (Novagen, USA), however, the recombinant protein was expressed only in Rosetta cells. A single colony of Rosetta cells containing pET construct was inoculated in 10 ml of Lysogeny Broth (LB) medium fortified with 50 µgml^−1^ kanamycin and the cells were grown overnight (O/N) at 37°C. 5 ml of this O/N grown culture was inoculated in 500 ml of LB medium containing kanamycin (50 µgml^−1^). The culture was grown in a shaker incubator with constant stirring till A600 reached ∼ 0.6. The culture was induced with 1.0 mM isopropyl-β-D-thiogalactopyranoside (Sigma, USA) and incubated at 25°C for 5 h. The bacterial cells were pelleted at 3000x*g* for 10 min at 4°C and resuspended in Buffer A (20 mM Tris-Cl pH 8.0, 250 mM NaCl, 10 mM imidazole). The cells were lysed in the presence of lysozyme (1 mgml^−1^) and Triton X-100 (0.1%) and the lysate was sonicated to reduce viscosity. The soluble fraction obtained after centrifugation was applied over a Ni-NTA agarose column (Ni^4+^ metal coupled agarose beads, Qiagen, USA) that was pre-equilibrated with Buffer A. The column was washed with Buffer B (20 mM Tris-Cl pH 8.0, 250 mM NaCl, 50 mM imidazole) and the recombinant protein was eluted with Buffer C (20 mM Tris-Cl pH 8.0, 250 mM NaCl, 250 mM imidazole). The recombinant *w*Bm-LigA protein appeared as a single band on a 10% sodium dodecyl sulfate (SDS) polyacrylamide gel electrophoresis (SDS-PAGE). The protein was dialyzed against Buffer B (20 mM Tris-Cl pH 8.0, 250 mM NaCl, imidazole 50 mM) and subsequently against Buffer D (Tris-Cl 20 mM, 250 mM NaCl). The protein content was estimated with Bradford reagent using Bovine serum albumin (BSA) as standard. Approximately ∼2 mg was recovered from 1 liter of the bacterial culture. For the Western blot analysis, purified recombinant protein from the SDS-PAGE gel was transferred to a nitrocellulose membrane. The membrane was blocked with 3% BSA for 1 h and incubated at room temperature (RT) with 1∶2500 dilution of mouse anti-His antibody (Sigma, USA), re-incubated with 1∶10,000 dilution of goat anti-mouse IgG-HRP antibody (Sigma, USA) for 1 h at RT and the blot was developed with the substrate 3,3′-diaminobenzidine tetra hydrochloride (DAB, Sigma, USA) [30].

### Phylogenetic analysis

Protein sequences of DNA ligases from the different organisms available on NCBI database were aligned using ClustalW software. The phylogenetic trees were constructed based on the protein sequence alignments with Maximum Likelihood (ML) Method using PROML programs of PHYLIP package version 3.69 with global rearrangements and randomized input order options. Bootstrap re-sampling was done with 100 replicates using the Seqboot program. Finally, the results from random datasets were summarized by consense that makes consensus trees by the Majority-rule consensus tree method. The phylogenetic trees were visualized using the Tree Drawing Tool-Drawgram.

### Structural Analysis

The CD spectra were recorded on JASCO J810 spectropolarimeter equipped with peltier temperature controller system in a 0.2 cm cell at 25°C. The measurements were carried out in 50 mM sodium phosphate buffer, pH 8.0 at a final protein concentration of 10 μM in the far-UV range (195 nm–240 nm). The ellipticity was reported as the mean residual ellipticity [θ] (10^3^deg.cm^2^dmol^−1^). K2D2 web server (http://www.ogic.ca/projects/k2d2/) was employed to estimate the ratio of secondary structure elements in the CD spectra [31]. On the other hand, the 2D structure prediction was also carried out using the GOR1V method (Garnier–Osguthorpe–Robson) available online at http://abs.cit.nih.gov/gor/. The 3D model of *w*Bm-LigA was predicted by amino acid homology modeling using 3D-JIGSAW automated web server (http://bmm.cancerresearchuk.org/~3djigsaw/) [32] and viewed by Rasmol (http://www.openrasmol.org/) [33].

### Fluorescence spectroscopy

The fluorescence spectra were recorded on a fluorescence spectrometer (Perkin Elmer LS50b) using an excitation wavelength of 280 nm and an emission wavelength in the range of 300–450 nm. The path length of quartz cell was 5 mm. The measurements were carried out at a final protein concentration of 1 μM in 50 mM sodium phosphate buffer, pH 8.0 at 25°C and *w*Bm-LigA was denatured with 6 M guanidine hydrochloride which served as control. For guanidine hydrochloride denaturation, the recombinant protein was dissolved in sodium phosphate buffer (50 mM, pH 8.0), incubated at 25°C O/N before measurements were made. This incubation time was sufficient for the reaction to achieve equilibrium under all denaturant concentrations.

### Nick closing activity and biochemical characterization of *w*Bm-LigA

Nick closing activity of *w*Bm-LigA was determined by the sealed duplex assay as described earlier [24] with minor modifications. This ligase assay format was based on ligation of a fluorescein labeled oligonucleotide to a biotinylated oligonucleotide within a double strand duplex hybridized on a streptavidin-coated microtitre plate. The amount of fluorescence captured following denaturantion of DNA duplex quantifies a successful ligation. Briefly, the three oligonucleotides viz. 5′-fluorescein AAAATGACCCC C-3′, 5′-CCAGACAACGTCG-biotin-3′and5′-GACGTTGTCTGGGGGGGGTCATTTT-3′ (Eurogentec, Germany) were suspended in Buffer H [5xSSC (0.75 M sodium chloride, 0.075 M sodium citrate solution), 0.1% BSA, 0.1% Triton X-100] and hybridized on Strepta Well HighBind plates (Thermoscientific, USA ) for 90 min at 20°C to produce nicked substrate. Wells were washed with 400 µl of ligase buffer (20 mM Tris-HCl, pH 8.0, 10 mM (NH_4_)_2_SO_4_, 10 mM MgCl_2_, 1.2 mM EDTA, and 1 mM dithioerythritol) and the ligation was performed for 60 min at 25°C in 200 µl of ligase buffer to which 5 nM of *w*Bm-LigA and 26 µM of NAD^+^ was added. This was followed by denaturation in 0.5% SDS, 0.5 M NaOH for 1 min. The fluorescence was measured at an excitation wavelength of 485 nm and an emission wavelength of 525 nm after washing the wells twice with 400 µl of TBS-T (Tris-buffer saline containing 0.05% Tween 20), and 0.1% BSA. Substrate concentration dependent nick activity curve of *w*Bm-LigA was generated for determining the steady-state Km for nicked substrate by measuring the formation of fluorescein labeled product at an increasing substrate concentration. To investigate the correlation between the concentration of enzyme and the substrate, different concentration of enzyme were mixed with a fixed amount of substrate such that enzyme, substrate concentration was in the ratio of 1, 0.04 or 0.02. The pH optima of *w*Bm-LigA was determined in Tris–HCl buffer at various pH range between 6.5 and 9.0 while the effect of temperature on the enzymatic activity was assessed between 0°C and 50°C. The effect of NAD^+^ cofactor on the nick activity was also observed between 0 μM and 30 μM concentrations of NAD^+^. To find out the effect of divalent cations on the enzymatic activity of *w*Bm-LigA, MgCl_2_, MnCl_2_, CaCl_2_, NiCl_2_ and CoCl_2_ were added at 10 mM concentration as the source of cations. The effect of monovalent ions on ligase activity was also investigated by carrying out reaction in the presence of NaCl, KCl and NH_4_Cl using the concentrations ranging between 0 mM and 200 mM. The effect of different concentrations of the chelating agent viz. EDTA was also determined on the nick closing activity.

### Cohesive end joining activity of *w*Bm-LigA and factors affecting this activity

For cohesive end joining activity of *w*Bm-LigA, the cohesive ends of lamda DNA digested with BstEII enzyme (New England Biolabs, UK) were used as a substrate. Standard reaction (20 µl) contained *w*Bm-LigA enzyme in 20 mM Tris-Cl (pH 8.0), 100 mM KCl, 10 mM MgCl_2_, 26 µM NAD^+^, 10 mM DTT, 500 ng of BstEII digested lamda DNA and the reaction was performed at 25°C for 60 min. The reaction was stopped by adding 4 µl of 50 mM EDTA followed by heating at 70°C for 5 min and subsequent chilling on ice for 5 min. The mixture was run on 1.0% agarose gel prepared in 1xTris acetate EDTA (TAE) buffer at 100 V for 90 min. The activity values were calculated by measuring the net intensity of the product band (1+4) relative to a non-substrate fragment (fragment 7). The band intensities were determined by measuring UV fluorescence of the bands using the volume rectangle tool of the Quantity One version 4 software package of ChemiDoc system (BioRad, USA), after capturing the digital images of the agarose gel [34]. The activity was observed at varying temperature (10°C–45°C), pH (6.5–8.5), enzyme concentration (0–25 nM) and NAD^+^ cofactor. The effect of different salts viz. MgCl_2_, MnCl_2_, CaCl_2_, NiCl_2_ and CoCl_2_ (as the source of divalent cations); and NaCl, KCl and NH_4_Cl (as the source of monovalent cations) was also determined on cohesive end ligation activity of *w*Bm-LigA. The time course of ligation of BstEII digested lamda DNA fragments into the product was observed by incubating the reaction mixture at different time intervals.

### Generation of polyclonal antibody to *w*Bm-LigA in BALB/c mice

Five eight-week-old male BALB/c mice were administered subcutaneously in the back region with 20 µg of *w*Bm-LigA protein mixed with Freund's complete adjuvant (FCA, Sigma, USA) in 100 µl volume. The animals further received two more booster doses of same amount of protein in Freund's incomplete adjuvant (FIA, Sigma, USA) on day 15 and day 21 post first immunization. The blood was collected from the animals just before immunization on day 0 for isolation of preimmune serum and on day 30 to isolate anti-*w*Bm-LigA serum.

### Presence of endogenous *w*Bm-LigA in adult worm, L3 and mf stages by immunoblotting

To find out the presence of *w*Bm-LigA in various life-stages of *B. malayi*, the crude extracts derived from adult worms, mf and L3 were run on 10% SDS-PAGE followed by Western blotting. The target protein was recognized by immunoblotting using polyclonal antibody to *w*Bm-LigA. For preparing the crude somatic extracts, ten adult worms (male+female), 1000 L3 and 1500 mf were homogenized in phosphate buffer saline (PBS) containing protease inhibitor cocktail (Sigma, USA), sonicated and supernatant was obtained after centrifugation at 12,000x g for 30 min. The supernatant was mixed with an equal volume of 2x sample buffer [Bromophenol Blue (10 mgml^−1^), SDS (4.4%), Tris-Cl (0.5 M), β- mercaptoethanol (300 mM)] and heated at 95°C for 5 min followed by separation onto the 10% SDS-PAGE. The separated proteins were then transferred to nitrocellulose membrane. The remaining steps were same as described above for localization of His tagged *w*Bm-LigA in Western blot except that the anti-*w*Bm-LigA antibody was used as a primary antibody (1∶500 dilution) instead of anti-His antibody. The recombinant *w*Bm-LigA purified through Ni-NTA column served as a positive control.

### Immuonolocalization of endogenous *w*Bm-LigA in *B. malayi* by confocal microscopy

To determine *w*Bm-LigA distribution in the parasites by immunolocalisation, the adult female worm and mf were fixed overnight in 4% paraformaldehyde in M9 buffer (42 mM Na_2_HPO4, 22 mM KH_2_PO4, 86 mM NaCl, 1 mM MgSO_4_.7H2O, pH 7.0) at 4°C and processed as described earlier [35]. Anti-*w*Bm-LigA antibody was used as a primary antibody (1∶700) while FITC conjugated IgG (1∶200 in 0.5% BSA) was used as a secondary antibody. The worms and mf were incubated with 4′,6′- diamidino-2-phenylindole (DAPI, 100 ngml^−1^) for 5 min before final washing with M9 buffer for staining the DNA. Parasites were mounted on a glass slide in 90% glycerol and 10% p-phenyenediamine (Sigma, USA) in the PBS. The parasites were observed and the images were captured with a Zeiss LSM 510 META (Zeiss, Jena, Germany) confocal laser scanning microscope equipped with a plan-apochromat 63X oil objective and 40X objective. For excitation of FITC-conjugated secondary antibody, argon laser lines of 488 nm were used. DAPI was excited at 405 nm. As a negative control, the same procedure was executed after treating parasites with BALB/c preimmune serum.

### Statistical analysis

Data were analysed with the help of statistical software GraphPad PRISM 3.0.
